# Disengaging Leadership Scale (DLS): Evidence of Initial Validity

**DOI:** 10.3390/ijerph18062824

**Published:** 2021-03-10

**Authors:** Irina Nikolova, Marjolein C. J. Caniëls, Wilmar Schaufeli, Judith H. Semeijn

**Affiliations:** 1 Department of Leadership and Organizational Behavior, BI Norwegian Business School, 0484 Oslo, Norway; 2 Faculty of Management, Open Universiteit, 6419 AT Heerlen, The Netherlands; marjolein.caniels@ou.nl (M.C.J.C.); judith.semeijn@ou.nl (J.H.S.); 3 Occupational & Organizational Psychology and Professional Learning, KU Leuven, 3000 Leuven, Belgium; wilmar.schaufeli@kuleuven.be; 4 Social, Health & Organizational Psychology, Utrecht University, 3508 Utrecht, The Netherlands; 5 Research Centre for Education and the Labour Market, Maastricht University, 6229 GT Maastricht, The Netherlands

**Keywords:** scale validation, disengaging leadership, engaging leadership, basic psychological needs

## Abstract

The main goal of this study was to develop a scale for measuring Disengaging Leader-ship (DEL) behaviors and to provide preliminary evidence for the validity of this new instrument. Developing such new measures is needed given current concepts that tap into negative leadership behaviors are rarely based on a sound theoretical framework. Drawing on the core premises of Self-Determination Theory (SDT) regarding employees’ basic needs and, more specifically, building on its more recent extended framework, including employees’ needs frustration, we derived four dimensions that constitute Disengaging Leadership behaviors (coercive disengaging leadership, isolating disengaging leadership, eroding disengaging leadership, and demotivating disengaging leadership). To examine the factor structure and psychometric properties of the new Disengaging Leadership Scale (DLS), Exploratory Factor Analysis (EFA), Confirmatory Factor Analysis (CFA), and reliability analyses were conducted. Results supported the hypothesized four-factor structure of the DLS and showed that this factorial structure remained invariant across employees occupying blue-collar, white-collar, or managerial positions. Finally, we successfully tested convergent, divergent, and construct validity of DLS. We established that DEL is associated with employees’ needs frustration and with their experiences of emotional exhaustion. It is concluded that the DLS has sound psychometric properties and can be used in future research on the dark side of leadership.

## 1. Introduction

Organizations nowadays rely largely on the skills and behaviors of their managers to shape an engaging and healthy work environment for their employees [1]. This is key as organizations can only benefit from their workforce if workers are happy, highly motivated, and actively striving to contribute to the success of the organization [2]. Accordingly, it is of utmost importance to foster and support employee motivation by, for instance, encouraging managers to help their subordinates fulfil their basic psychological needs (BPN) at work [3,4]. Yet, despite best intentions, managers sometimes fail to fulfil employees’ BPNs (i.e., the need for autonomy, competence, connectedness, and meaningfulness) [4] or even actively thwart them [5,6,7]. Such management behaviors can have dire consequences [7,8], as previous studies indicated that frustration of basic psychological needs [5,6] can be detrimental for employees’ functioning at work and can lead to ill-being [5,6,9]. Considering the key role leaders have in fulfilling employees’ basic psychological needs [4], we draw upon the theoretical framework of basic psychological needs [3] and needs thwarting [6], and propose a new leadership concept—Disengaging Leadership (DL)—that taps into four leadership behaviors that can thwart employees’ BPNs.

Recent research on its counterpart, engaging leadership [4,10,11]—a concept based on the BPNs satisfaction framework [3]—indicates that a positive leadership style that is aimed at fulfilling employee’s basic needs, does in fact fulfil basic needs, and may lead to positive outcomes for employees. As in recent years, researchers have, on the one hand, acknowledged the conceptual difference between the *absence* of positive leadership behaviors (i.e., lack of active supportive behaviors that might fail to fulfil the BPNs) and the *presence* of negative leadership behaviors (i.e., undermining and destructive acts towards followers that can thwart employees’ BPNs) [12]; and, on the other hand, have expressed critique that the majority of the existing leadership concepts are not based on solid theoretical reasoning (e.g., [13]), the need of a concept that taps into leader’s demotivating and disengaging behaviors that is rooted in a well-acknowledged theory (e.g., BPN thwarting) has become clear.

Studies that investigate negative leadership traits and behaviors, typically assess the overall negative outcomes (e.g., depression, emotional exhaustion, performance decline) of these behaviors; some of the most frequently studied negative leadership traits and behaviors are abusive leadership [14,15,16], bullying practices [17], narcissism among chief executive officers [18], dark triad [19,20], and destructive leadership [21]. Although these studies demonstrate the devastating outcomes of a negative leadership style, they rarely provide a sound theoretical rationale that can help understand how leadership behaviors undermine employee motivation (by, for instance, thwarting the fulfilment of their BPNs).

To date, only a handful of studies have attempted to link negative leadership behaviors to need satisfaction [7,8]. However, these studies typically focused on one general or a few narrowly-defined negative behaviors (e.g., abusive leadership [7]) which hampers a more rigorous examination of the effects of discrete leadership behaviors on employee motivation. Despite initial evidence, to date little is known about the specific demotivating or disengaging behaviors leaders might engage in that may thwart employees’ BPN fulfilment. Our limited knowledge on how different leadership behaviors correspond to the thwarting of each basic psychological need was the main reason for developing and validating the Disengaging Leadership Scale (DLS). Accordingly, building on the work of Bartholomew et al. [5,6] and borrowing its theoretical underpinnings from the BPNs Theory (BPNT [3]), the current contribution identifies four leadership behaviors that induce psychological needs thwarting in employees.

Developing and validating a multidimensional diagnostic instrument that can tap into Disengaging Leadership has also practical implications. An assessment tool for measuring disengaging leadership may help to create awareness about disengaging leader behaviors and the extent to which leader actions may thwart employees’ BPNs, instead of supporting them. Such awareness is key for fostering work environments where occupational health and psychological safety are valued and carefully fostered. Our instrument may also help human resource management professionals to quickly and accurately identify the aspects of leader behavior that require improvement, for instance, by training or coaching. In this way, our measures may support organizations in promoting and nurturing employee motivation and well-being. By enabling scholars and practitioners to gain insights into these issues, the purpose of our study aligns with the goals of the current special issue on Non-Technical Perspectives for Improving Safety in the Workplace, namely, to promote better understanding of the precursors of a healthy and safe work context. Evidently, workplaces that strive to foster psychologically safe environments, would need a reliable measure to detect the disruptive leadership behaviors that might harm employee psychological health.

### 1.1. Disengaging Leadership

Over the past two decades considerable attention was devoted to negative leadership behavior, also dubbed the dark side of leadership [18,22,23]. Studies have underscored the conceptual difference between the absence of effective leadership behaviors and leadership behaviors that explicitly include destructive aspects [12]. The latter refer to leader’s acts that are generally perceived as harmful towards the followers and the organization [24]. These acts can either be physical or verbal, active or passive, direct or indirect [23,24]. Leaders may actively undermine subordinates, for example, by engaging in bullying practices [17], being abusive [14], lying, and acting in ways that can directly jeopardize the health of their subordinates [25].

In the current study we unravel the container notion of “negative leadership” by focusing on discrete leader behaviors that might thwart BPNs. We draw on BPNT [3] to develop a leadership concept that focuses on leader behavioral patterns that can frustrate, instead of satisfy, employees’ BPNs. Exploring a suchlike leadership style can foster our understanding of the processes that evolve from need frustration. Previous contributions [6] suggested that any need that is being frustrated leads to alternative, self-protective, psychological accommodations (i.e., the development of controlling regulatory styles, compensatory motives, or need substitutes) that over time may cause further thwarting and result in poor well-being [3].

We define disengaging leadership as leader behaviors that frustrate employees’ BPNs for autonomy, competence, connectedness, and meaningfulness, thereby vexing their motivation and work engagement. Accordingly, we distinguish four dimensions that tap into disengaging leadership behaviors.

First, disengaging leadership behavior frustrates employees’ needs for autonomy. This need pertains to the extent to which individuals feel in control and responsible for their own behavior. A leader can frustrate the subordinate’s need for autonomy by, for instance, enforcing work methods and tasks in a way that offers no room for the employee’s own ideas and ways of working. Second, disengaging leadership behaviors frustrate employees’ need for competence. The need for competence refers to the extent to which people feel effective in their interactions with their direct social environment and experience appreciation by others for their skills and abilities. A leader can frustrate the subordinate’s need for competence by, for instance, denying access to development opportunities and undermining self-efficacy. For example, leaders may give employees the feeling that they are not capable of doing their work properly, or solving complicated situations.

Third, disengaging leader behaviors actively thwart employees’ need for connectedness. This need concerns the degree to which individuals experience meaningful relationships with others and feel that they are accepted in and belong to the social environment [3]. A leader can frustrate the subordinate’s need for relatedness by, for instance, instigating distrust and sabotaging collaborations (e.g., suggesting that one cannot count on other colleagues).

Finally, disengaging leader behaviors frustrate the need of employees to experience meaningfulness in their work. This need refers to the degree to which individuals experience their work as meaningful and contributing to some larger goal [4]. A leader can frustrate the subordinate’s need for meaningfulness and accomplishment by, for instance, downplaying the impact of the work itself, or giving the feeling that the work is useless (e.g., suggesting that one’s work is of little or no value for the organization or society).

### 1.2. The Four Dimensions of the DLS

Disengaging leadership behaviors may trigger powerful emotional responses among the affected employees which may cause a narrowing of the individual’s thought–action repertoire (broaden-and-build theory [26]) and result in more negative perceptions of the work context and ultimately in ill-health. Below, we elaborate on the four dimensions that constitute Disengaging Leadership (DEL) and on how they may affect employees.

#### 1.2.1. Coercive Disengaging Leadership (Need for Autonomy Frustration)

Coercive leadership is characterized by leadership behaviors that are aimed at actively controlling different aspects of employee functioning at work. Such behaviors go beyond the lack of supporting employee autonomy [27], they actively deprive employees from decision latitude by instigating the leader’s own desires and will (i.e., they regard their wishes and ideas to be superior to those of their subordinates). Subsequently, when describing the conditions that specify the frustration of the need for autonomy, we draw on studies that examine leaders’ over-controlling behaviors (e.g., [6,28]). For instance, studies have identified micro-managing and over-controlling as some of the most destructive behaviors a leader can engage in, because these behaviors deprive employees from decision latitude and flexibility when conducting their daily tasks [28].

Autonomy deprivation can have various detrimental consequences, including apathy and alienation [29]. Whereas autonomy-supportive environments are generally considered to be conducive of individual’s proactivity and to foster personal autonomy, competence, and relatedness [30], contextual factors, such as coercive leadership, that threaten the individual’s autonomy by inflicting preconceived views and preferred behaviors on others, can impede perceptions of one’s own self-efficacy and meaningfulness [6].

As the need for autonomy is assumed to represent the individuals’ need to behave according their own interests and values [29,31], autonomy threats may be deeply disruptive because they may jeopardize the individual’s perceived chances for achieving their personal goals and may cause a conflict between the imposed (by the leader) actions and the individual’s beliefs. Under such circumstances, employees’ feeling of volition and responsibility for their own behavior may be strongly inhibited, because their basic need for autonomy is frustrated.

#### 1.2.2. Eroding Disengaging Leadership (Need for Competence Frustration)

Eroding leadership is characterized by leadership behaviors that are aimed at the obstruction of employee professional development and diminishing their professional efficacy, or sense of competence. Such leadership behaviors deprive employees from access to learning opportunities and erode employees’ sense of professional efficacy by systematically pointing out one’s weaknesses and demonstrating mistrust in, and lack of confidence in, one’s competences.

Inherent to the need for competence is that individuals need to feel effective in their daily interactions with others at work and to perceive opportunities to master their professional skills [3]. Typically, competence-supportive environments and leadership behaviors are aimed at providing positive feedback and incentives for learning, acknowledging individuals’ accomplishments and expressing belief in individuals’ ability to achieve their goals. Conversely, competence-thwarting environments and leadership behaviors emphasize individuals’ professional and personal weaknesses and instigate feelings of low competence and of inability to improve [32].

Altogether, competence-thwarting leadership behaviors, erode employees’ sense of mastery and self-efficacy in handling their tasks, and undermine their belief that they can successfully acquire new competences (preventing individuals from, for instance, experimenting with new work methods or taking on novel tasks) [32,33]. Eroding leadership can be particularly harmful not just because it denies employees the opportunity to excel in their current jobs, but also because it might impact their perceived employability. In addition, it may trigger chronic feelings of impoverished learning efficacy (causing employees to question their personal and professional capacities to change and improve).

#### 1.2.3. Isolating Disengaging Leadership (Need for Relatedness or Connectedness Frustration)

Isolating leadership is characterized by leader behaviors that are aimed at disconnecting employee from the rest of the team. Such leaders engage in behaviors that would weaken or corrupt employee’s professional relationships with other colleagues by instigating mutual distrust and disliking (i.e., isolation from the team).

A disengaging, isolating leader hinders employees from establishing effective and harmonious professional relationships by distancing himself or herself from the employee and by discouraging an employee’s attempts to build close connections with others at work. In its essence, the need for relatedness pertains to the individual’s need to be emotionally connected to others (i.e., to care for and being cared for by other individuals) and to belong to a group or community [34]. Isolating disengaged leadership, therefore, refers to the continuous, active obstruction of an employee’s needs for connectedness and efforts to bond with others. When the need for connectedness is frustrated, for instance by social exclusion, individuals show impaired self-regulation [35] and reduced cognitive performance [36]. Reportedly, the satisfaction of this need is key for the internalization of work-related appeals, rules and regulations; and in contexts characterized by secure relatedness, the internalization process is more likely to be successful [32].

The isolating disengaging leadership dimension captures the way in which relationships between co-workers are destabilized by certain leadership behaviors, thereby thwarting their need for connectedness. In the leadership literature, leadership behaviors that disrupt or actively sabotage the relationships between colleagues were not left unnoticed. For instance, [14] alludes to this aspect of leadership by asking respondents about the extent in which their direct supervisor makes negative comments about them to others, or does not allow them to interact with their co-workers. In a similar vein, Rocchi and colleagues describe connectedness-thwarting behaviors as “being distant with others, not connecting emotionally, excluding them, not listening, and not being available when needed” [31] (pp. 424).

#### 1.2.4. Demotivating Disengaging Leadership (Need for Meaningfulness Frustration)

Demotivating leadership is characterized by leadership behaviors that are aimed at creating, among employees, an image and sense that their job is meaningless and their work does not contribute to anything important. Such behaviors make an employee’s work effort seem meaningless by not recognizing it, or downplaying employee’s contributions.

This dimension is inspired by recent studies [4,37] that demonstrated the presence of a fourth basic psychological need at work, namely the need for meaningfulness [38,39]. Meaningfulness is defined as “the desire to be engaged in activities that are useful, important, significant, and are in line with one’s personal values” [4] (pp. 4). Research has supported the idea that the need for meaningfulness is well-positioned among the basic psychological needs, because of its strong motivational and well-being-supportive properties [4,37]. Moreover, the study of Rahmadani and colleagues [4], provided empirical evidence by showing that the need for meaningfulness belongs to the set of BPN. Specifically, the need for meaningfulness was shown to be positively related to work engagement, and this association was established in addition to the effects of the other three basic psychological needs.

Because meaningfulness is considered to be derived from activities that are important to the individual and congruent with one’s personal values [40,41] thwarting this basic need implies that the individual’s values and sense of contribution and accomplishment might be jeopardized. Researchers have argued that the sense of having a prosocial impact, and feeling that your work matters is key to the individual’s occupational identification [42] and well-being [43].

### 1.3. Convergent, Divergent and Construct Validity of the DLS: Theoretical Underpinnings

In order to establish the convergent validity of the DLS, the relationships of the proposed instrument with other variables in the nomological network were investigated. Convergent validity is demonstrated when the new scale correlates with conceptually similar measures [44]. To establish convergent validity, we used the abusive leadership scale from Tepper [14]. We selected the instrument of Tepper for three main reasons. First, the abusive leadership concept and the items used in the scale tap into a broad range of negative leadership behaviors, some of which (single items) assessing leader actions akin to those in the four dimensions of our disengaging leadership scale. For instance, our eroding disengaging leadership dimension focuses on competence obstruction by the leader, which aspect has been touched upon by the Tepper’s instrument as well (e.g., “My leader tells me that I am incompetent”). Additionally, our isolating disengaging leadership dimension that assesses employees’ deprived connectedness due to their leader’s actions, is measured by Tepper (e.g., “My leader makes negative comments about me to others” or “My leader does not allow me to interact with my coworkers”). Second, the items in the Tepper’s scale are short, clearly worded and easy to understand, which makes them user friendly. Third, this scale was often used in prior studies and was proven to be a valid instrument—e.g., [45,46,47]. Abusive leadership measures the conscious harmful behavior of a leader towards a follower, thereby undermining the follower’s effective functioning and work pleasure [16]. Abusive leadership is expected to be positively related to disengaging leadership as it taps into the same kind of negative leader behavior, yet the abusive leadership measure is not systematically addressing the thwarting of BPNs. We expect that:

**Hypothesis** **1.** *Abusive leadership is highly positively related to the DLS.*

To further assess convergent validity, we used the engaging leadership (EL) scale [10]. The engaging leadership scale (ELS) is built on the concept of needs satisfaction (with four dimensions that tap into Engaging Leadership—empowering leadership, strengthening leadership, connecting leadership, and inspiring) in a similar way as our DLS is built on the concept of needs frustration. For each of the four ELS dimensions, our DLS has an antipode. As the DLS was constructed independently from the ELS, DLS items do not exactly mirror opposite ELS items. Furthermore, both constructs are conceptually different in the sense that the absence of engaging leadership behaviors geared towards need satisfaction does not inherently imply that leaders actively frustrate subordinates’ psychological needs, i.e., the presence of disengaging leadership behaviors. Therefore, we expect that:

**Hypothesis** **2.** *Each of the dimensions of DLS is negatively related to each of the ELS dimensions (a). Moreover, there is a stronger relationship between each matching pair of dimensions of DEL and EL (i.e., between Coercive DEL and Empowering EL, Eroding DEL and Strengthening EL, Isolating DEL and Connecting EL, Demotivating DEL and Inspiring EL) (b).*

Divergent validity is demonstrated when the proposed new construct shows low associations with a theoretically unrelated (or weakly related) construct [44]. We chose the concept of perceived mobility, which indicates the degree to which an employee expects to be able to find another job. Studies have shown that negative leadership behaviors are associated with higher rates of absenteeism and turnover [14], but the relationship between negative leadership behaviors and perceived mobility can go in two directions. On the one hand, negative leadership behaviors can undermine self-esteem of employees [23], thereby decreasing their perceived ability to find another job. On the other hand, negative leadership behaviors may also motivate employees to leave a bad situation and hence stimulate their perception of being able to change jobs. Whereas some causal relationship between leadership behaviors and perceived mobility may be expected, experiencing disengaging leadership, which is a contextual factor, is theoretically different from perceiving to be able to switch jobs, which is strongly related to one’s personal resources. Therefore we expect:

**Hypothesis** **3.** *a weak, non-significant relationship between disengaging leader behaviors and perceived mobility.*

The construct validity of the DLS was further examined by investigating the associations of the four dimensions of DLS with two important and theoretically relevant employee outcomes: needs frustration and emotional exhaustion. Prior studies have documented severe effects of negative leadership behaviors, as such behaviors cause employees’ emotional exhaustion (e.g., [15,48]) hinder overall needs satisfaction [8], and instigate an intention to quit [49]. Therefore, we expect that the four dimensions measuring the different behaviors that are set to frustrate employees’ needs would relate positively to needs frustration and subsequently to emotional exhaustion.

**Hypothesis** **4.** *Each of the dimensions of DLS is positively and significantly related to each of the four basic psychological needs frustrations (a). Moreover, there is a stronger relationship between each matching disengaging leadership dimension and its corresponding psychological need frustration (i.e., between Coercive DEL and autonomy frustration, Eroding DEL and competence frustration, Isolating DEL and connectedness frustration, Demotivating DEL and meaningfulness frustration) (b).*

## 2. Method

### 2.1. Participants and Procedure

The study’s sample used for the analyses consisted of 400 employees working in different organizations. An international online research company operating in Bulgaria collected the data. Invitations were sent out at the end of November 2017 to 4300 individuals; the respondents were given 10 days to complete the questionnaire. Participation in the survey was on a voluntary basis and respondents could discontinue (drop out) their participation at any point. The respondents were informed that the data will be used to for scientific research and their personal data, such as names, phone numbers, or e-mail addresses are not made accessible for the researchers at any point. From the people who were invited to participate in the survey, 731 clicked on the link, however, 109 of the 731 did not finish the survey (i.e., closed it before filling the entire questionnaire), which implies a 15% drop out. Their age ranged from 19 to 64 years (*M* = 39.18 years; *SD* = 11.27 years); in total, 49.8% of the respondents were female and 90.8% were employed with a permanent contract. With regard to their occupational level, 19.5% were unskilled blue-collar workers, 20.3% were skilled blue-collar workers or foreman, 16.8% lower-level white-collar workers, 22.8% occupied an intermediate white-collar or supervisory (of a white-collar employees) function, 14.0% were upper white-collar worker or middle management, and 6.8% were managers or directors. In the sample, people working in different occupational groups were included (production, logistics, sales, health, education and training, research, just to name few).

### 2.2. Item Generation

We generated the initial set of items for measuring disengaged leadership based on a literature review on deviant leadership behaviors, need thwarting and need satisfaction—e.g., [6,10,14,28,50]. We strived to create a scale that can be applied in different occupational settings and professional groups. Our choice to develop a four-dimensional instrument was guided by the four BPNs. An initial item pool of 53 items was formulated by the first and third author of the current study, and subsequently subjected to discussion with two experts in leadership and test-construction research. In addition, we invited 10 experts employed at the Work and Organizational Psychology department of a Belgium university. They were asked to critically evaluate of each dimension of the DLS in writing; specifically focusing on, (1) whether the definition clearly describes the specific leadership behaviors related to the matching need frustration, (2) whether the proposed dimensions correctly reflect the need frustration as described by self-determination theory, (3) to assess the items based on the clarity of the formulation and on their suitability to measure the disengaging leadership behaviors as defined for each dimension, and (4) to add critical feedback. As requested, all 10 experts returned a document with their evaluations and comments. We carefully read and systematized their feedback and based on this expert evaluation, we selected the final items that were included in the questionnaire. As a result, each of the four dimensions of the DLS was measured with five items each. In the questionnaire, the items for measuring DLS were introduced with the following text: “To what extent do the following statements apply to your current work situation?” Responses could be given on a five-point Likert scale ranging from 1 (fully disagree) to 5 (fully agree).

### 2.3. Measures

We tested the DLS’s construct, convergent and divergent validity using several theoretically relevant constructs. Unless otherwise indicated, a 5-point response scale was used ranging from 1 (strongly disagree) to 5 (strongly agree).

Engaging leadership (a four-dimensional concept) was measured with 12 items developed by Rahmadani and colleagues [4]. A sample item per dimension is: “My supervisor makes me feel like I contribute to something important” (inspiring leadership; Cronbach’s α = 0.94); “My supervisor encourages me to develop my talents as much as possible” (strengthening leadership; Cronbach’s α = 0.91); “My supervisor makes sure I feel at home working with my team” (connecting leadership; Cronbach’s α = 0.86); “My supervisor encourages me to give my own opinion” (empowering leadership; Cronbach’s α = 0.92).

Abusive leadership was measured with a 15-item scale developed by Tepper [14]. A sample item is: “My supervisor does not allow me to interact with my colleagues”. The total scale’s internal consistency was very high (Cronbach’s α = 0.96).

Perceived mobility was measured with two items developed by Tepper [14]. A sample item is: “I would have no problem finding an acceptable job if I quit” (Cronbach’s α = 0.86).

Need frustration (a four-dimensional concept) was measured with 16 items. Items for autonomy frustration, competence frustration, and connectedness frustration were adapted from the instrument developed by Bartholomew et al., [5]. In this study, we incorporated a fourth aspect of need frustration—frustration of the need for meaningfulness (measured with four items); in doing so, we followed the example of Rahmadani and colleagues, who argued that the need for meaningfulness is key (and can be measured by using three items). A sample item per dimension is: “I feel prevented from making choices with regard to the way I conduct my work” (autonomy frustration; Cronbach’s α = 0.91); “There are situations where I am made to feel inadequate” (competence frustration; Cronbach’s α = 0.89); “I feel other people dislike me” (connectedness frustration; Cronbach’s α = 0.88); “People around me believe that I make no contribution” (meaningfulness frustration; Cronbach’s α = 0.94).

Emotional exhaustion was assessed with a four-item scale from the Burnout Assessment Tool [51]. A sample item is “At work, I feel mentally exhausted”. Cronbach’s for this scale was good (α = 0.86).

## 3. Results

### 3.1. Exploratory and Confirmatory Factor Analysis

To explore the factorial structure of the DLS we first conducted an Exploratory Factor Analyses (EFA) followed by a Confirmatory Factor Analysis (CFA). Whereas EFA was carried out using the statistical package SPSS 24 (IBM, Armonk, NY, USA), CFA was performed by means of MPlus 7 (Muthén & Muthén, Los Angeles, CA, USA) [52].

The results of the EFA (maximum likelihood extraction with oblique rotation) indicated that DLS has a two-factor structure with one factor including the items of the Coercive Disengaging Leadership dimension (7.23% explained variance), and another dominant factor incorporating all other items (66.90% explained variance).

In a next step, to explore further the factorial structure of the scale, we tested and compared five competing models. First, Model 1 (a one-factor model) was constructed to test whether all items measure one general DEL factor. Second, Model 2 was designed to test the two-factor structure that emerged from the EFA (i.e., Coercive DEL as one factor, and another DEL factor incorporating all other items). Third, an alternative two-factor model (Model 3) was analyzed, with one factor including all items intended to measure Coercive and Eroding DEL (because the items in these two dimensions tap into cognitive and task-related aspects), and a second factor incorporating the items intended to assess Isolating and Demotivating DEL (because the items in these two dimensions assess rather affective aspects related to feeling socially included, acknowledged, and contributing in a meaningful way). Fourth, a three-factor model (Model 4) was constructed with factor 1—Coercive DEL, factor 2—Isolating DEL, and factor 3 included all items of the Eroding and Demotivating DEL dimensions. This model was constructed to test whether the items from the Eroding and Demotivating DEL dimensions might cluster together because individuals who are let to believe that they are not competent to carry out their daily tasks, might experience their work as fruitless and deprived of meaning. Finally, these alternative models were compared to the hypothesized four-factor model (Model 5) where each factor represents one of the four dimensions of DEL (Coercive DEL, Isolating DEL, Eroding DEL, and Demotivating DEL).

Goodness-of-fit [53] was evaluated by means of several fit indices: chi-square (χ^2^), root-mean-square errors of approximation (RMSEA ≤ 0.08), standardized root-mean-square residual (SRMR ≤ 0.08), comparative fit index (CFI ≥ 0.90), and Tucker–Lewis index (TLI ≥ 0.90).

In Table 1, the fit indices and the χ^2^ difference tests of the five alternative models are presented. The one-factor model (Model 1) had poor fit (χ^2^ (*df* = 211) = 3130.79, RMSEA = 0.19, SRMS = 0.26, CFI = 0.71, TLI = 0.69), whereas the two-factor model (Model 2, which reflects the dimensions as indicated by EFA) showed a significantly better fit (Δχ^2^(42) = 158.74, *p* < 0.001). However, the fit indices for Model 2 indicate an unsatisfactory fit as well (χ^2^ (*df* = (169) = 1543.40, RMSEA = 0.14, SRMS = 0.05, CFI = 0.85, TLI = 0.83). In a similar vein, the proposed second two-factor model (Model 3) showed a significantly better fit compared to the one-factor model (Δχ^2^(42) = 157.52, *p* < 0.001); yet, the fit indices for Model 3 also indicate a poor fit to data (χ^2^ (*df* = 169) = 1555.60, RMSEA = 0.14, SRMS = 0.06, CFI = 0.85, TLI = 0.83). Next, the three-factor model (Model 4) fitted the data relatively well (χ^2^ (*df* = 167) =1003.49, RMSEA = 0.12, SRMS = 0.05, CFI = 0.91, TLI = 0.90) and had a significantly better fit than the two-factor model (Model 2). Last, the hypothesized four-factor model (Model 5) showed the best fit to the data (χ^2^ (*df* = 164) = 656.60, RMSEA = 0.09, SRMS = 0.04, CFI = 0.95, TLI = 0.94). This model fitted the data significantly better than the three-factor model (Δχ^2^(3) = 34.69, *p* < 0.001). Factor loadings of Model 5 ranged from 0.64 to 0.87 for Coercive DEL, from 0.76 to 0.86 for Eroding DEL, from 0.90 to 0.94 for Isolating DEL, and from 0.86 to 0.95 for Demotivating DEL.

In conclusion, the results from the CFA demonstrated that the theoretically derived four-factor structure of the DLS is empirically supported by the data. Moreover, Model 5 showed a better fit than any other of the four alternative models. Finally, each of the four dimensions of the DEL scale showed good reliability; Cronbach’s alpha was 0.90 (Coercive DL), 0.92 (Eroding DL), 0.97 (Isolating DL), and 0.96 (Demotivating DL), respectively. Table 2 presents all DLS items (as intended to measure the four DEL dimensions—Model 5), and their factor loadings.

### 3.2. Invariance Testing

To establish if the structure of the DLS is invariant across sub-samples a multi-group comparison was carried out. The measurement invariance analyses were performed using three sub-samples. First, the sample was split according to occupational level: blue-collar workers (n_1_ = 159); white-collar workers (n_2_ = 158); and managers (n_3_ = 83). By means of multigroup CFA (estimation method: maximum likelihood) we tested whether the proposed four-factor scale was invariant across these three sub-samples by restricting and comparing the model fit in several subsequent steps resulting in tests of weak, strong, and strict measurement invariance [54]. A decrease in the CFI greater than 0.01 indicated a meaningful decrement in fit [55]. We started by estimating the configural invariance of our scale. To this end we analyzed an unconstrained model where all parameters were freely estimated across the three groups (χ^2^(492) = 1291.641, *p* < 0.001, CFI = 0.916, TLI = 0.90, AIC = 15150.09, BIC = 15940.40, RMSEA = 0.11, SRMR = 0.05). Subsequently, we constrained the factor loadings of the respective factors to be equal across the three groups, which allowed us to test for metric invariance (χ^2^(524) = 1332.22, *p* < 0.001, CFI = 0.915, TLI = 0.91, AIC = 15126.67, BIC = 15789.25, RMSEA = 0.11, SRMR = 0.05). Results showed that the model with constrained factor loadings did not show a significant decrease in CFI compared to the unconstrained model (∆CFI < 0.01), which indicates that the metric invariance held equal across the three sub-samples. Next, we set the factor loadings and intercepts to be equal across the three sub-samples (χ^2^(556) = 1370.86, *p* < 0.001, CFI = 0.91, TLI = 0.914, AIC = 15101.31, BIC = 15636.17, RMSEA = 0.11, SRMR = 0.06); also, this did not worsen the model fit significantly (∆CFI < 0.01) hence providing support for the scalar invariance of the DLS. Based on these results (as the subsequent tests did not lead to a significant loss of fit), conventional levels of measurement invariance were established for the three occupational groups.

### 3.3. Convergent, Divergent, and Predictive Validity

In Table 3, the means, standard deviations, inter-correlations, and reliabilities of the study variables are presented. We further explored the validity of the DLS, by scrutinizing the correlations between the DLS and several criterion variables. More specifically, we examined the correlations between the four dimensions of DLS and abusive leadership, and between DLS and the four dimensions of Engaging Leadership (EL) to test for convergent validity. The results obtained from the correlation analyses (Table 3) align with the expectation that each of the dimensions of DLS will be highly positively and significantly related to abusive leadership (correlations ranged from 0.61 to 0.73, *p* < 0.001). Therefore, Hypothesis 1 was confirmed.

In line with our expectations, all four dimensions of our second criterion variable—EL correlated negatively and significantly with the sub-scales of the DLS (Table 4); the absolute values of coefficients ranged from 0.30 to 0.55. Specifically, the correlation between Coercive DEL and Empowering EL (the two constructs based on the need for autonomy frustration vs satisfactions) was negative and significant (*r* = −0.41; *p* < 0.001); the correlation between Eroding DEL and Strengthening EL (the two constructs based on the need for competence frustration vs satisfactions) was negative and significant (*r* = −0.45; *p* < 0.001); the correlation between Isolating DEL and Connecting EL (the two constructs based on the need for connectedness frustration vs satisfactions) was negative and significant (*r* = −0.52; *p* < 0.001); the correlation between Demotivating DEL and Inspirational EL (the two constructs based on the need for meaningfulness frustration vs satisfactions) was negative and significant (*r* = −0.55; *p* < 0.001).

As visible from Table 4, even though all dimensions for DEL and EL are negatively and significantly correlated among each other (*r* ranging from 0.49 to 0.80, *p* < 0.001), only for two of the four DEL dimensions, the correlations confirm the expected matching pattern with the corresponding EL dimensions (i.e., Eroding DEL had the highest correlation with Strengthening EL, and Demotivating DEL with Inspiring EL). Therefore, while the results confirmed Hypothesis 2a, Hypothesis 2b was rejected.

In line with our expectations, none of the correlations between the four dimensions of DEL and the criterion variable for divergent validity—mobility—were significantly correlated. The absolute values of the correlation coefficients ranged from 0.00 to 0.04. Therefore, Hypothesis 3 was confirmed.

Associations of DLS with other theoretically relevant constructs were investigated. We tested the associations between the four sub-scales of the DLS and employees’ needs frustration and emotional exhaustion. As expected, all dimensions of DLS were positively and significantly related to the four dimensions of needs frustration with correlations ranging from 0.32 to 0.63. Specifically, the correlation between Coercive DEL and the need for autonomy frustration was positive and significant (*r* = 0.63; *p* < 0.001); the correlation between Eroding DEL and the need for competence frustration was positive and significant (*r* = 0.49; *p* < 0.001); the correlation between Isolating DEL and the need for connectedness frustration was positive and significant (*r* = 0.43; *p* < 0.001); the correlation between Demotivating DEL and the need for meaningfulness frustration was positive and significant (*r* = 0.57; *p* < 0.001).

As can be seen from Table 3, even though all dimensions for DEL and the four basic needs frustration are positively and significantly correlated among each other (*r* ranging from 0.49 to 0.80, *p* < 0.001), only for two of the four DEL dimensions, the correlations confirm the expected matching pattern with the corresponding need frustration (i.e., Eroding DEL had the highest correlation with the frustration of need for competence, and Demotivating DEL with the frustrated need for meaningfulness). Therefore, while the results confirmed Hypothesis 4a, Hypothesis 4b was rejected.

In addition, and as expected, the four dimensions of DLS were positively and significantly related to emotional exhaustion (coefficients ranging from 0.32 to 0.39).

In addition to these analyses, we constructed and tested a mediation model (using structural equation modeling in MPlus). We conceived the mediation as follows: DEL -> needs frustration -> emotional exhaustion. Initially, we specified the model using first-order latent factors for each of the constructs (i.e., DEL was represented by each of the four dimensions as separate latent factors, need frustration was represented by four latent factors, and exhaustion by only one); however, considering the small sample size, it was not possible for MPlus to execute the analyses. Alternatively, we created a more parsimonious model and increased the degrees of freedom, by specifying DEL as a second-order construct; the CFA for the second-order DEL construct showed good fit (RMSEA = 0.09, SRMR = 0.04, CFI = 0.94, TLI = 0.93) albeit slightly worse than the CFA for the original first-order DEL scale (RMSEA = 0.09, SRMR = 0.04, CFI = 0.95, TLI = 0.94). Specifying DEL as a second-order construct allowed us to test the proposed mediation model, which showed an acceptable fit (RMSEA = 0.08, SRMR = 0.09, CFI = 0.90, TLI = 0.89). The results indicated that DEL related positively and significantly to each of the four need frustration concepts (autonomy frustration β = 0.65, *p* < 0.001; competence frustration β = 0.67, *p* < 0.001; connectedness or relatedness frustration β = 0.61, *p* < 0.001; meaningfulness frustration β = 0.65, *p* < 0.001). However, only one of the four aspects of need frustration—the autonomy frustration—related significantly to emotional exhaustion (β = 0.25, *p* < 0.001), while the other facets were not significantly associated with the outcome variable (competence frustration β = 0.09, *p* = 0.33; connectedness or relatedness frustration β = 0.06, *p* = 0.45; meaningfulness frustration β = 0.13, *p* = 0.14). Additionally, DEL showed no significant relationship with emotional exhaustion (β = 0.13, *p* = 0.11).

### 3.4. Demographics

Next, to explore whether the demographic characteristics in our sample have the propensity to affect how employees experience DEL, we regressed gender, age, hours of work per week, and occupational level on each of the four dimensions of DLS. Results (Table 5) showed that age was positively and significantly associated with each of the four dimensions of DL. Specifically, age was positively and significantly related to Coercive DEL (β *= 0*.19, *p =* 0.001), Eroding DEL (β *= 0*.14, *p =* 0.001), Isolating DEL (β *= 0*.19, *p =* 0.001), and Demotivating DEL (β *= 0*.17, *p =* 0.001). In addition, gender showed a negatively though marginally significant relation with Coercive DEL (β *=* −0.10, *p =* 0.05), indicating that men might be more susceptible to the negative influence of Coercive DEL. However, gender was not significantly related to any of the other dimensions of DEL. Last, with regard to employees’ occupational level, the regression coefficients indicated that employees occupying lower positions reported more Isolating DEL (β *=* −0.11, *p =* 0.03).

## 4. Discussion and Conclusions

In this study, we developed and validated a new concept that taps into disengaging leader’s behaviors. The concept of disengaging leadership is based on the notion that certain set of leader’s behaviors may frustrate the needs of the subordinates. The concepts need frustration and need satisfaction stem from the self-determination theory (SDT [3]), which maintains that humans strive towards growth and integrity when their basic needs (for autonomy, competence, and connectedness) are being met. Alternatively, defensiveness or reduced functioning may occur when individuals are exposed to a controlling, critical, or rejecting social context because such an environment may thwart or frustrate their needs [56].

Corresponding to the three types of basic human needs according to SDT [29] and in line with recent studies [4] that propose a fourth BPN—meaningfulness—we developed four matching dimensions of DEL. Each of these dimensions reflects leader behaviors that frustrate a specific core human need. Accordingly, each dimension of DEL was labeled using an adjective that summarizes the leadership style the dimension represents.

To investigate the robustness of our newly developed instrument, we conducted a series of analyses that are typically used in psychometric testing of new scales. Results showed that DLS is a reliable instrument with Cronbach’s alpha values for each of the four dimensions considerably above the recommended in literature threshold of 0.70. Results from a series of CFAs indicated that the four-factor model of DLS, where each item loaded on the intended factor, had a very good fit, and that its fit was significantly better in comparison with other models (i.e., models with one, two, and three-factor structures). Hence, the notion that the four dimensions of the DLS—coercive disengaging leadership, isolating disengaging leadership, eroding disengaging leadership, and demotivating disengaging leadership—each represent a distinct factor was empirically supported.

Cross-validation analyses, where occupational level (blue-collar, white-collar or management positions) was used as a criterion to split the sample, supported factorial invariance of the four-factor model of the DLS across the three occupational groups. This implies that our instrument measures the same concept in each of the three groups.

We examined the DLS for convergent, divergent and predictive validity. We used the Abusive Leadership scale and the four-dimensional construct Engaging Leadership (EL; empowering, strengthening, connecting, and inspiring leadership) to test for convergent validity. Results supported the hypothesized high positive relationship between Abusive Leadership and each of the dimensions of DEL (Hypothesis 1). Additionally, all four dimensions of the criterion variable were strongly and negatively related to each of the sub-scales of the DLS. In line with our initial idea, the established relationships were, strongly and negatively (Hypothesis 2a), yet not too strongly (e.g., above 0.70) related to each other, indicating that while the DEL and EL constructs tap into two opposing leadership styles, they do not represent the exact opposites of the same construct. In addition, we found no perfect matches between the dimensions of DEL and EL (Hypothesis 2b not confirmed). This indicates that while to some extent we can find a connection between the dimensions based on the specific need, there is an overarching leadership concept that may account for the high correlations between all dimensions of the DEL and EL concepts. In addition, although we distinguish between four basic needs, these needs are highly inter-correlated, which might explain why clear matches between the dimensions are not found. This demonstrates the convergent validity of our psychometric instrument.

We used the construct job mobility (Hypothesis 3) to test the divergent validity of DLS. Results showed that the four dimensions of the DLS are psychometrically distinct from the construct job mobility. Job mobility was unrelated to the four dimensions of DEL (i.e., Coercive DEL, Isolating DEL, Eroding DEL, and Demotivating DEL). In all, based on these results the divergent validity of the DLS vis-à-vis job mobility was supported.

Finally, in keeping with our rationale that DEL as a particularly destructive type of leadership will frustrate employees’ basic needs, and therefore might cause ill-being, we found positive associations between the DLS and employees’ needs frustration and emotional exhaustion. As expected, the four dimensions of DLS showed moderate to strong positive relationships with the four dimensions of needs frustration (Hypothesis 4a), indicating that these two concepts are theoretically and content related. Yet, only for two of the four DEL dimensions, the correlations confirmed the predicted higher relationship with the matching need frustration (i.e., Eroding DEL had the highest correlation with the frustration of need for competence, and Demotivating DEL with the frustrated need for meaningfulness). This indicates that, while to some extent specific DEL dimensions frustrate the corresponding need, because these needs are highly intercorrelated, clear matches between the dimensions cannot be found (Hypothesis 4b not confirmed). Additionally, the four dimensions of DLS were positively and significantly linked to employees’ reduced well-being (i.e., increased emotional exhaustion).

In sum, alongside providing support for the theoretical assumptions about the DLS’s structure, our findings indicate the importance of diagnosing and monitoring disengaging leadership behaviors in order to safeguard employees’ occupational well-being and to prevent hazards for employee motivation because of their growing frustration from not having their basic needs met. The current study provides initial evidence on the good psychometric properties of the DLS and hinted at potentially relevant relationships with other constructs, such as emotional exhaustion and needs frustration, whereby potentially inspiring future research to further explore how DEL (with its four dimensions) affect other well-being and motivational outcomes (e.g., flow and engagement experiences, job satisfaction, intrinsic, and extrinsic motivation).

### Limitations, Strengths, and Implications of the Study

A total of three limitations of this contribution are worth mentioning. First, the cross-sectional nature of the study curbed the possibility to test the predictive validity of DLS. Therefore, we could not test the directionality of the theoretically suggested relationships (i.e., DEL as a trigger of needs frustration, and of emotional exhaustion). Although leadership is considered one of the most powerful sources of influence on employees’ well-being and general psychological states [57,58], and despite ample research evidence on the negative effect of deviant leadership behaviors on employee attitudes, behavior, and well-being [23,59], one could argue that employees who experience sub-optimal psychological states and ill-being are more likely to view their work environment (which includes their leader) as less positive. Yet, it seems unlikely that employees perturbed psychological needs and well-being will raise negative perceptions of the leader’s behaviors to an extent that would explain perceptions of disengaging leadership. In addition, the DLS taps into specific behaviors the leader engages in, which, we believe, steer employees’ attention to the evaluation of more observable (i.e., more objective) indicators of leadership style, limiting the chance of reversed causal effects. We encourage future studies to provide a cross-lagged investigation of the relationship between DEL and employee motivational and well-being outcomes to help empirically substantiate (or repudiate) this claim.

Second, in this study we used self-reports to measure employee perceptions of the leadership style of their managers and of the characteristics of their job, which may induce common method variance [60]. Even though one could argue that such perceptions are best surveyed among the employees, because they are the direct object of the manager’s leadership behaviors which could impact employees’ reactions and well-being, still, the inclusion of other methods of data collection such as evaluations from third parties (e.g., team members or other managers evaluating the leadership behaviors of their colleague) can strengthen our evidence, as these methods have additional value over the self-reports.

Third, the relatively small sample size of the study (*N* = 400) might potentially have affected the study findings causing an underestimation of relationships when examining the different types of validity. Additionally, testing for measurement invariance by splitting the sample into three groups further reduced the sample size for each group, which might also have affected our findings by reducing the statistical power. Therefore, the evidence from the stability testing of our measure using cross-validation should be considered with some caution. Future studies could provide a more rigorous examination of our instrument by using a larger sample and employing a longitudinal design. The latter will allow to study the predictive validity of our scale, by examining cross-lagged relationships between DLS and pertinent employee outcomes such as, just to name few, need thwarting, a-motivation, intentions to leave, and absenteeism. Furthermore, using a larger sample for the invariance testing with a sample split based on education, gender, sector, or selecting two sub-samples obtained at two time points, could add to the current initial evidence of the psychometric properties of DLS.

Also, two notable strengths of the current contribution warrant attention. First, we developed a new leadership scale, rooted in one of the most prominent motivational theories—SDT ([29]; and in particular building on its more recent extended framework explaining employees’ needs frustration), which scale measures disengaging behaviors of the leader. The majority of the existing scales on leadership styles do not originate from a specific theoretical framework, instead they seem to emerge from a general idea about the individual characteristics that are assumed to constitute certain leadership style. This is why in the past decade, some of the most widely used leadership concepts such as transformational leadership faced strong criticisms about lacking clear definition, clear (not overlapping) factorial structure, and being unsound [13].

Second, the DLS can be used as a diagnostic tool for evaluating employees’ relationships with their leader and for monitoring for leader’s behaviors that might be experienced as disengaging. By testing the instrument’s invariance among groups of employees working at different occupational levels (i.e., blue-collar, white-collar, or management positions), we demonstrated that DLS is easy to understand (i.e., does not include complex item wording or profession-specific jargons) and can be successfully used among employees of different occupational groups. Furthermore, owing to the multidimensional structure of the DLS, we can elucidate on the different aspects (i.e., specific behaviors) of the leader that constitute disengaging leadership, and how distinct leader’s behaviors can impact specific employee attitudinal, behavioral, or well-being outcomes.

Given that the disengaging leadership concept, to an extent, mirrors the idea of engaging leadership (reflecting the leader’s contribution to the satisfaction of the four basic needs of employees), future studies might wish to examine the theoretical assumptions these concepts build upon. Testing a conceptual model that examines the causal paths between, on the one hand, each of the disengaging leadership dimension, the corresponding frustration of the four needs, and employee outcomes (e.g., occupational strain and counterproductive behaviors), and on the other hand, each of the engaging leadership dimensions, the satisfaction of the four needs, and employee positive outcomes (e.g., engagement) might be particularly valuable for further theory development. In addition, echoing the recommendation from a recent meta-analytic study on narcissism in CEOs [18], we propose that future scholarly work could focus on the antecedents of DEL to better understand the underlying motivations and processes that unlock DEL behaviors, relying on the theoretical lenses from personality theory. Understanding how personality traits (e.g., dark triad; [20,61]) shape DEL behaviors, and how these behaviors affect employees’ psychological needs and their work-related experiences can be very valuable.

To aid Human Resources practitioners in dealing with deviant leader’s behaviors (e.g., DEL behaviors) in their organizations, future scholarly work may focus on identifying potential contextual factors that can buffer the negative impact of DEL on employee functioning. Additionally, studying the effectiveness of managerial training programs that might potentially help disengaging managers flip side their negative behaviors and learn new ways of interacting with their employees (e.g., in a way that helps employees fulfill their basic psychological needs) might be valuable for organizations.

By developing a new instrument that measures leaders’ behaviors which can lead to needs frustration and subsequently to emotional exhaustion among employees, the current study contributes to the overall theme of the current special issue “Occupational Safety and Health”. Advancing knowledge on the inhibitors of need satisfaction, and providing preliminary insights into the potential detrimental impact of DEL on e’ployees’ psychological health might be valuable to scholars and practitioners who are interested in the “Non-Technical Perspectives for Improving Safety in the Workplace”, the topic of the current special issue.

## Figures and Tables

**Table 1:** Fit Indices of Competing Nested Factor-models, Standardized Maximum Likelihood Estimates (*N* = 400).

Model	N. Factors	χ^2^	df	RMSEA	SRMR	CFI	TLI	Model Comparison	|Δχ^2^|
Model 1	1-factor model	3130.79	211	0.19	0.26	0.71	0.69		
Model 2	2-factor model	1543.40	169	0.14	0.05	0.85	0.83	Model 1–Model 2	158.74 ***
Model 3	2-factor model	1555.60	169	0.14	0.06	0.85	0.83	Model 1–Model 3	157.52 ***
Model 4	3-factor model	1003.49	167	0.12	0.05	0.91	0.90	Model 3–Model 4	55.21 ***
Model 5	4-factor model	656.60	164	0.09	0.04	0.95	0.94	Model 4–Model 5	34.69 ***

Note: Model 1 = all items loaded on one factor; Model 2 = 2 factor model with Coercive Disengaging Leadership (DEL) + one general DEL factor (with all the rest of the items); Model 3 = 2 factor model with factor 1 Coercive and Eroding DEL + factor 2 Isolating and Demotivating DEL; Model 4 = 3 factor model with factor 1 Coercive DEL + factor 2 Isolating DEL and factor 3 including Eroding and Demotivating DL; Model 5 = 4 factor model with each factor representing each of the four proposed dimensions of DL; Goodness-of-fit indices abbreviations stand for: χ2 = chi-square, RMSEA = root-mean-square errors of approximation, SRMR = standardized root-mean-square residual, CFI = comparative fit index, TLI = Tucker–Lewis index; *** *p* < 0.001.

**Table 2:** Items of the Disengaging Leadership Scale (*N* = 400; Model 5).

Dimension	Items	Factor Loadings
Coercive Disengaging Leadership (frustration need for autonomy)	Pressures me to do my job in a specific way	0.64
	Enforces work methods which I would not choose myself	0.80
	Instigates his or her vision without asking about my opinion	0.87
	Enforces his or her ideas without taking my opinion into account	0.87
	Burdens me with tasks which are against my personal convictions	0.80
	Obstructs my professional development	0.84
Eroding Disengaging Leadership (frustration need for competence)	Denies me access to trainings and courses at work	0.76
	Ensures I do not get any interesting tasks form which I can learn new things	0.84
	Gives me the feeling that I am not capable of doing my job well	0.86
	Suggests that I cannot solve complicated situations at work	0.84
	Sabotages smooth collaborations between my colleagues and myself	0.90
Isolating Disengaging Leadership (frustration need for connectedness)	Tries to create divisions between me and my colleagues	0.94
	Let’s me know that I cannot count on my colleagues	0.90
	Creates conflicts between me and my colleagues	0.94
	Instigates distrust between me and my colleagues	0.93
	Downplays the importance of my work	0.86
Demotivating Disengaging Leadership (frustration need for meaningfulness)	Gives me the feeling that my work is useless	0.91
	Makes me feel like my work does not matter to anyone or anything	0.95
	Suggests that my work is of little or no value for our organization	0.92
	Let me know that what I do is pointless and unimportant	0.92

Note: All items above were preceded by the statement ‘My direct supervisor’.

**Table 3:** Means (M), Standard Deviations (SD), Intercorrelations, and Reliabilities (in Parentheses) of the Study Variables (*N* = 400).

Scale	*M*	*SD*	1	2	3	4	5	6	7	8	9	10	11	12
1. Coercive DEL (aut)	2.70	0.94	(0.90)											
2. Eroding DEL (comp)	2.26	0.91	0.75 **	(0.92)										
3. Isolating DEL (connec)	2.11	0.97	0.64 **	0.81 **	(0.97)									
4. Demotivating DEL (mean)	2.12	0.96	0.66 **	0.82 **	0.84 **	(0.96)								
5. Engaging Leadership	3.45	0.89	−0.42 **	−0.53 **	−0.51 **	−0.57 *	(0.97)							
6. Abusive Leadership	1.65	0.82	0.61 **	0.68 **	0.70 **	0.73 **	−0.42 **	(0.96)						
7. Mobility	3.48	0.97	−0.04	0.01	0.00	−0.01	0.07	0.01	(0.86)					
8. Needs frustr: autonomy	2.94	0.94	0.63 **	0.54 **	0.48 **	0.55 **	−0.43 **	0.56 **	−0.01	(0.91)				
9. Needs frustr: competence	2.63	0.91	0.48 *	0.49 **	0.44 **	0.56 **	−0.41 **	0.55 **	−0.07	0.68 **	(0.89)			
10. Needs frustr: connect.	2.24	0.82	0.43 **	0.48 **	0.43 **	0.50 **	−0.31 **	0.43 **	−0.05	0.50 **	0.66 **	(0.88)		
11. Needs frustr: meaning	2.16	0.86	0.44 **	0.51 **	0.44 **	0.57 **	−0.32 **	0.50 **	−0.05	0.49 **	0.66 **	0.80 **	(0.94)	
12. Emotional Exhaustion	2.83	0.89	0.39 **	0.37 **	0.32 **	0.36 **	−0.33 **	0.36 **	0.00	0.44 **	0.41 **	0.36 **	0.37 **	(0.86)

Note: DEL = Disengaging Leadership. In parentheses the corresponding need (or frustration thereof) is indicated: aut = need for autonomy, comp = need for competence, connec = need for connectedness, mean = need for meaningfulness. Engaging Leadership represents a one-dimensional scale here; this is to avoid an over-sized table with individual correlations for each dimension of the concept; an overview of the correlations between each of the Engaging Leadership and DEL dimensions is provided in Table 4. * p < 0.05; ** p < 0.01. M = means; SD = standard deviation

**Table 4:** Means, Standard Deviations, Intercorrelations, and Reliabilities (in Parentheses) of the Disengaging and Engaging Leadership dimensions (*N* = 400).

Scale	*M*	*SD*	1	2	3	4	5	6	7	8
1. Coercive DEL (aut)	2.70	0.94	(0.90)							
2. Eroding DEL (comp)	2.26	0.91	0.75 **	(0.92)						
3. Isolating DEL (connec)	2.11	0.97	0.64 **	0.81 **	(0.97)					
4. Demotivating DEL (mean)	2.12	0.96	0.66 **	0.82 **	0.84 **	(0.96)				
5. Empowering EL (aut)	3.54	1.01	−0.41 **	−0.50 **	−0.47 **	−0.54 **	(0.92)			
6. Strengthening EL (comp)	3.45	0.91	−0.30 **	−0.45 **	−0.40 **	−0.40 **	0.81 **	(0.91)		
7. Connecting EL (connec)	3.49	0.90	−0.36 **	−0.48 **	−0.52 **	−0.54 **	0.79 **	0.84 **	(0.86)	
8. Inspiring EL (mean)	3.29	1.04	−**0.**46 **	−0.51 **	−0.48 **	−0.55 **	0.83 **	0.81 **	0.83 **	(0.94)

Note: DEL = Disengaging Leadership is presented by four dimensions (coercive, eroding, isolating, demotivating), EL = Engaging Leadership is presented by four dimensions (empowering, strengthening, connecting, inspiring). In parentheses the corresponding need (or frustration thereof) is indicated: aut = need for autonomy, comp = need for competence, connec = need for connectedness, mean = need for meaningfulness; the correlations between the matching (based on the psychological needs) DEL and EL dimensions are presented with bold ** p < 0.001.

**Table 5:** Summary of Regression Analyses: Standardized Regression Coefficients (*N* = 400).

Demographics	Disengaging Leadership Dimensions			
	Coercive DEL (aut)	Eroding DEL (comp)	Isolating DEL (connect)	Demotivating DEL (mean)
Gender	−0.10 *	−0.10	−0.05	−0.06
Age	0.19 ***	0.14 **	0.19 ***	0.17 ***
Hrs work per week	0.01	−0.02	0.02	−0.05
Occupational level	−0.01	−0.07	−0.11 *	−0.10
				
F (df )	5.38(4) ***	3.70(4) **	5.06(4) **	4.09(4) **

Note: *** p < 0.001, ** *p* < 0.01; * p < 0.05; 1 = Male, 2 = Female.

## Data Availability

The data presented in this study are available on reasonable request from the corresponding author.
